# Idiopathic central precocious puberty in a Klinefelter patient: highlights on gonadotropin levels and pathophysiology

**DOI:** 10.1186/s12610-020-00117-1

**Published:** 2020-12-09

**Authors:** Salwan Maqdasy, Bertrand Barres, Gaelle Salaun, Marie Batisse-Lignier, Celine Pebrel-Richard, Kelvin H. M. Kwok, André Labbé, Philippe Touraine, Florence Brugnon, Igor Tauveron

**Affiliations:** 1 CHU Clermont-Ferrand, Service d’endocrinologie, diabétologie et maladies métaboliques, 58, rue Montalembert, F-63003 Clermont-Ferrand, France; 2 Université Clermont Auvergne, Faculté de médecine, F-63003 Clermont-Ferrand, France; 3 Laboratoire GReD, Université Clermont Auvergne, F-63003 Clermont-Ferrand, France; 4 Centre Jean Perrin, Service de Médecine nucléaire, F-63003 Clermont-Ferrand, France; 5 CHU Clermont-Ferrand, service de cytogénétique médicale, F-63003 Clermont-Ferrand, France; 6 Department of Biosciences and Nutrition, Karolinska Institutet, 141 83 Stockholm, Sweden; 7 CHU Clermont-Ferrand, Service de pédiatrie, F-63003 Clermont-Ferrand, France; 8 Hôpital Pitié-Salpêtrière, service d’endocrinologie et médecine de la reproduction, Centre de maladies endocriniennes rares de la croissance et du développement, Paris, France; 9 Assistance Médicale à la Procréation, CECOS, CHU Clermont-Ferrand, F-63000 Clermont-Ferrand, France; 10 Université Clermont Auvergne, INSERM, U1240 Imagerie Moléculaire et Stratégies Théranostiques, CHU Clermont-Ferrand, F-63000 Clermont Ferrand, France

**Keywords:** Precocious puberty, Idiopathic, Klinefelter, Gonadotropin, Puberté précoce, idiopathique, Klinefelter, gonadotrophines

## Abstract

**Background:**

Idiopathic central precocious puberty (ICPP) is supposed to be non-existent in a context of testicular destruction that is typically present in Klinefelter syndrome (KS). Herein, we describe a rare case of ICPP in a Klinefelter patient (47,XXY) with 2 maternal X chromosomes. Moreover, we highlight the differences in gonadotropin levels in comparison to males with ICPP and a normal karyotype.

**Case presentation:**

An 8 years old boy with a history of cryptorchidism was evaluated for precocious puberty (Tanner staging: P2/G3). Both testes measured 25x35mm. His hormonal profile confirmed a central origin of precocious puberty with high serum testosterone (4.3 ng/ml), luteinizing hormone [LH (3.5 UI/l)] and follicle stimulating hormone [FSH (7.7 UI/l)] levels. Luteinizing hormone-releasing hormone (LHRH) test amplified LH and FSH secretion to 24 and 14 UI/l respectively. Brain magnetic resonance imaging (MRI) was normal. No MKRN3 mutation was detected. He was treated for ICPP for two years. During puberty, he suffered from hypergonadotropic hypogonadism leading to the diagnosis of KS (47,XXY karyotype). Chromosomal analysis by fluorescent multiplex polymerase chain reaction (PCR) using X chromosome microsatellite markers identified 2 maternal X chromosomes.

Analysing 8 cases of KS developing ICPP (our reported case and 7 other published cases) revealed that these KS patients with ICPP have higher LH and FSH levels during ICPP episode than in ICPP patients with a normal karyotype (ICPP with KS vs ICPP with a normal karyotype: LH levels 9.4 ± 12 vs 1.1 ± 0.6 UI/l; FSH levels 23.1 ± 38.5 vs 2.7 ± 1.5 UI/l). Furthermore, their response to gonadotropin-releasing hormone (GnRH) stimulation is characterized by excessive LH and FSH secretion (LH levels post-GnRH: 58 ± 48 vs 15.5 ± 0.8 UI/l; FSH levels post-GnRH: 49.1 ± 62.1 vs 5.7 ± 3.9 UI/l).

**Conclusions:**

ICPP in boys is extremely rare. The pathophysiology of ICPP in KS is unknown. However, maternal X supplementary chromosome and early testicular destruction may play a significant role in the initiation of ICPP, in part explaining the relative *“overrepresentation* of ICPP in KS. Thus, karyotype analysis could be considered for boys suffering from ICPP, especially if testicular size is smaller or gonadotropins are significantly elevated.

**Supplementary Information:**

The online version contains supplementary material available at 10.1186/s12610-020-00117-1.

## Résumé

**Introduction:**

Une puberté précoce centrale idiopathique (PPCI) est. extrêmement rare chez le garçon. Sa prévalence chez les garçons présentant un syndrome de Klinefelter (SK) est méconnue. Nous décrivons un cas rare de PPCI chez un garçon présentant un SK (47,XXY). De plus, nous surlignons les différences observées entre les taux de gonadotrophines en cas de PPCI chez les patients avec caryotype normal ou présentant un syndrome de Klinefelter.

**Cas clinique:**

Un garçon de 8 ans avec des antécédents de cryptorchidie a été pris en charge pour une puberté précoce (PP) (Stade de *Tanner*: P2/G3). Les diamètres des testicules étaient élevés pour son âge (25x35mm pour chaque côté). Le bilan hormonal a confirmé une PP avec des concentrations sanguines de testostérone, LH et FSH élevées à 4.3 ng/ml, 3.5 UI/l et 7.7 UI/l respectivement. Un test à la LHRH a révélé une réserve très élevée de LH (24 UI/l) et de FSH (14 UI/l). Aucune lésion tumorale n’a été mise en évidence par l’IRM. La recherche de mutation du gène MKRN3 n’a révélé aucune anomalie. Le garçon a été suivi et pris en charge pour une PPCI durant 2 ans. A la fin de la puberté, un hypogonadisme périphérique a permis d’évoquer le diagnostic de SK (47,XXY). L’analyse chromosomique par PCR en utilisant des marqueurs de chromosome X a confirmé l’origine maternelle des deux chromosomes X.

**Discussion:**

À ce jour, 7 cas de PPCI ont été décrits chez des garçons avec un SK. Nous avons analysé les données de ces patients et comparé aux données de patients avec une PPCI mais avec un caryotype normal. Nous mettons en exergue un taux significativement élevé de la LH et de la FSH lors du diagnostic de la PPCI chez les patients avec un SK en comparaison avec un caryotype normal (LH: 9.4 ± 12 vs 1.1 ± 0.6 UI/l; FSH: 23.1 ± 38.5 vs 2.7 ± 1.5 UI/l). De plus, les taux de la LH et FSH après une stimulation par la GnRH étaient également très élevés (LH: 58 ± 48 vs 15.5 ± 0.8 UI/l; FSH: 49.1 ± 62.1 vs 5.7 ± 3.9 UI/l).

**Conclusions:**

Une PPCI est. extrêmement rare chez le garçon. La description de 8 cas de PPCI dans la littérature indique un surrisque de cette pathologie chez les garçons souffrant d’un SK. La physiopathologie reste méconnue. L’origine maternelle du chromosome surnuméraire et la destruction précoce du testicule en lien avec l’anomalie chromosomique pourraient favoriser une puberté précoce. Ainsi, une analyse du caryotype pourrait être proposée chez un garçon souffrant d’une PPCI.

## Background

Puberty in boys is initiated when gonadotropin-releasing hormone (GnRH) secretion is amplified, inducing the secretion of gonadotropins. Pulsatile secretion of luteinizing hormone (LH) and follicle stimulating hormone (FSH) is accompanied by enlarged testicular volume, testosterone secretion and initiation of spermatogenesis [1]. The age of puberty initiation is variable across sex, ethnicity, and is influenced by genetic and nutritional factors. Precocious puberty (PP) is generally due to premature sexual steroid secretion [1–4]. In most cases, puberty is considered precocious in boys if it is initiated before 9 years old. The prevalence of PP is about 1 to 8/10000 with a female/male ratio of 9/1. Central precocious puberty (CPP) is caused by earlier pulsatile secretion of GnRH and gonadotropins [2]. Unlike delayed puberty, CPP remains idiopathic in most of the cases (ICPP) [4]. Many observations suggest a possible link between chromosomal anomalies and PP, especially in Klinefelter syndrome (KS) [5–7]. KS is the most frequent dysgonosomia in boys that leads to incomplete puberty and hypergonadotropic hypogonadism [8]. Indeed, most cases of PP in KS arise from germ cell tumors secreting human chorionic gonadotropin (hCG). While many cases of ICPP in KS have been reported in the literature, [9–14], the physiopathology remains unclear. Furthermore, the hormonal profile of patients with ICPP and KS is not well characterized. The study of patients with divergent problems such as ICPP and KS might improve the understanding of ICPP pathophysiology and point out factors that influence the timing of puberty initiation.

We describe a case of ICPP in a boy who, a few years later, developed hypogonadism in his adulthood secondary to KS (with 2 maternal X chromosomes). In addition to our patient, we analyze 7 other cases of ICPP in KS patients previously reported in the literature. Among patients with ICPP, those who are also suffering from KS have higher levels of gonadotropins and an exaggerated response to GnRH stimulation compared to those with a normal karyotype. This comparison highlights the possibility of a primitive testicular factor, such as precocious germ cell and Leydig cell dysfunction, participating in the induction of ICPP in KS.

## Case presentation

A boy, born in 1994, was evaluated for suspicion of precocious puberty. At 8 years old, his height (150 cm) exceeded the mean for age by 3 standard deviations with a growth rate of 10 cm per year. He developed puberty signs (Tanner staging: P2/G3). Both testes measured 25x35mm, the penis was enlarged and increased in length. Bone age was advanced (10 years according to modified Sempé’s method). His past history was marked by cryptorchidism, operated at age of 1 year. No facial dysmorphia, behavioural abnormalities or mental retardation were noticed.

His hormonal profile confirmed a central origin of precocious puberty with high serum testosterone (4.3 ng/ml) for age, and detectable LH (3.5 UI/l) and FSH (7.7 UI/l) (adult male reference values for FSH: 1.1–9.9 UI/l, and LH: 0.9–6.6 UI/l).

Luteinizing hormone-releasing hormone (LHRH) test amplified LH and FSH secretion to 24 and 14 UI/l respectively (Table 1). Hypothalamic-pituitary MRI was normal. Due to advanced bone age and growth acceleration, he was treated with triptoreline (3 mg, intramuscular injection every 28 days). Under this treatment, LH, FSH and testosterone were attenuated to a prepubertal level (0.2 UI/l, 0.4 UI/l and 0.2 ng/ml respectively) (Table 1). In 2005, the treatment was stopped, and puberty resumed. At age of 20, he consulted for hypogonadism and bilateral gynecomastia. His definitive height was 178 cm (predicted height was 173 cm, with mid-parental height estimated to 166 cm) and his weight was 86 kg (+ 1 SD). The testicular size was reduced (25 × 12 mm). Further evaluation confirmed azoospermia, and low testosterone levels (1.16 ng/ml, reference values for adults > 2.75 ng/ml) with hypergonadotropic hypogonadism (LH and FSH increased to 26 and 40 UI/l respectively) (Table 1). Testicular biopsy revealed a hyalinized tissue with failed spermatozoa retrieval.

**Table 1:** Clinical and biological parameters of the patient during and after puberty

Age	8 years	*9.5 years*	*9 years*, *10 months*	*10 years*, *4 months*	11.5 years	20 years
LH (UI/l)	3.5	*0.2*	*0.2*	*0.2*	0.3	26
FSH (UI/l)	7.7	*0.4*	*0.4*	*0.4*	0.5	39.6
Testosterone (ng/ml)	4.35	*0.3*	*0.2*	*0.2*	0.3	1.16
Testicular diameter (mm)	35 × 25	*35 × 20*	*35 × 20*	*35 × 20*	35 × 20	25 × 12
Tanner staging	P2G3	*P2G3*	*P2G3*	*P2G3*	P3G3	P3G2
Bone age	9 years, 11 months		*11 years, 6 months*	*11 years*, *6 months*	12 years, 3 months	

LH: luteinizing hormone; FSH: follicle stimulating hormone; UI: International units

Period of Triptoreline treatment**.** Reference values: FSH: 1.1–9.9 UI/l, LH: 0.9–6.6 UI/l

Chromosome analysis was performed on GTG-banded and RHG-banded metaphases prepared from cultured peripheral blood according to the standard protocols.

The origin of the supernumerary X chromosome was investigated by fluorescent multiplex polymerase chain reaction (PCR) to study short tandem repeats polymorphisms (supplementary methods)**.**

Peripheral blood cytogenetic analysis of our patient revealed 47 chromosomes, with an extra X chromosome in all analyzed metaphases, confirming KS. Array-comparative genomic hybridization (CGH) excluded the presence of any other genomic imbalance, except for copy number variations (CNV) previously described in the normal population according to the Database of Genomic Variants (http://projects.tcag.ca/variation). Microsatellite analysis in X chromosome showed two X chromosomes of maternal origin. Furthermore, no mutation was found in the coding regions of *MKRN3* gene.

An informed written consent was obtained from the patient and his parents to perform the genetic analyses and to accept the publication of the data in this article.

## Literature analysis and discussion

We collected and analyzed clinical and hormonal data in our case and 7 other cases of ICPP in KS reported in the literature [9–14]. The clinical and biological data at definitive puberty in each case were also considered. The characteristics of the 8 cases of ICPP in KS are summarized in Table 2**.** We also compared the hormonal profile between ICPP patients with KS (8 cases) and those with a normal karyotype reported in the literature (the most comprehensive cohort is published by Pigneur et al. [15]). Compared to patients with a normal karyotype (46,XY), KS patients have significantly higher LH and FSH levels during precocious puberty (KS + ICPP vs normal karyotype+ICPP: LH levels 9.4 ± 12 vs 1.1 ± 0.6 UI/l; FSH levels 23.1 ± 38.5 vs 2.7 ± 1.5 UI/l). Moreover, the response to LHRH in KS patients is more drastic (KS + ICPP vs normal karyotype+ICPP: LH levels post-LHRH 58 ± 48 vs 15.5 ± 0.8 UI/; FSH levels post-LHRH: 49.1 ± 62.1 vs 5.7 ± 3.9 UI/l) (Table 3). Even higher basal- and stimulated- LH and FSH levels during ICPP were noted in a KS patient with 48, XXYY karyotype [12] compared to boys with either normal karyotype [15] or 47,XXY (7 patients) (Table 2).

**Table 2:** Analysis of characteristics of Klinefelter patients with ICPP

Characteristics during precocious puberty							Final puberty-adulthood				
Studies	Karyotype	Age at PP	LH	LH post LHRH	T	TV	Height (cm)	LH	FSH	T	TV
Leon et al. [9]	**Mosaic XY/** **XXY/XXXY**	**8**	**–**	**–**	**–**	**–**	**173**	**–**	**–**	**–**	**–**
Mühlendahl et al. [10]	**47,XXY**	**8.3**	**6.3**	**42**	**3**	**3.4**	**181**	**–**	**–**	**–**	**3**
Mühlendahl et al. [10]	**47,XXY**	**8**	**–**	**–**	**1.1**	**5**	**189**	**15**	**39**	**0.1**	**8**
Bertelloni et al. [11]	**47,XXY**	**5**	**4.2**	**37**	**2.8**	**4**	**176**	**16**	**13**	**2.9**	**4**
Bertelloni et al. [12]	**48,XXYY**	**7**	**31**	**130**	**4**	**–**	**176**	**45**	**130**	**4.2**	**–**
Fryns et al. [13]	**47,XXY**	**–**	**–**	**–**	**–**	**–**	**–**	**–**	**–**	**–**	**–**
Gonzales-Ellis et al. [14]	**47,XXY**	**8.5**	**1.9**	**–**	**0.86**	**–**	**164**	**3.5**	**–**	**2.7**	**4**
Reported case	**47,XXY**	**8**	**3.5**	**24**	**4.35**	**6**	**178**	**19**	**22**	**1.16**	**4**
**All (Mean ± SD)**		**7.5 ± 1.3**	**9 ± 12**	**58.2 ± 48**	**2.7 ± 1**	**4.6 ± 1**	**177 ± 7**	**20 ± 17**	**51 ± 61**	**2.2 ± 1.4**	**4.6 ± 1.7**

*FSH* Follicle stimulating hormone, *LH* Luteinizing hormone, *LHRH* Luteinizing hormone-releasing hormone, *PP* Precocious puberty, *T* Testosterone levels, *TV* Testicular volume. Testosterone levels (T) are in ng/ml. TV corresponds to testicular volume in ml. LH and FSH are in UI/l.

**Table 3:** Gonadotropin levels in patients with ICPP with a normal karyotype (adapted from *Pigneur* et al. [15]) and ICPP in cases with KS

	LH (UI/l)	LH Post-GnRH (UI/l)	FSH (UI/l)	FSH Post-GnRH (UI/l)	T (ng/ml)
ICPP (*Pigneur* et al. *2008)* (28 patients) (Mean ± SD)[15]	1.1 ± 0.6	15.5 ± 0.8	2.7 ± 1.5	5.7 ± 3.9	2.6 ± 2.1
ICPP+KS (8 cases) (5–9 years) (Mean ± SD) Review of cases in the present study	9.4 ± 12	58 ± 48	23.1 ± 38.5	49.1 ± 62.1	3.1 ± 1.3

*ICPP* Idiopathic central precocious puberty, *KS* Klinefelter syndrome, *FSH* Follicle stimulating hormone, *LH* Luteinizing hormone, *GnRH* Gonadotropin-releasing hormone, *SD* Standard deviation, *T* Testosterone levels. Data are presented as mean ± SD

In our patient, despite the precocious puberty, target height was attained. Similarly, in other reported cases of ICPP with KS, the height was attained in all patients, with (3 patients including our case) [9, 10] or without anti-androgen treatment (5 patients). This is explained by the potential role of short-stature homeobox gene (SHOX) overexpression in such patients due to their dysgonosomia compensating for an eventual short stature risk related to PP [16]. However, testosterone levels at mid-puberty (14–15 years) and the testicular volume in these ICPP patients are identical to the classical puberty profile of KS [17–20].

Indeed, around 450 boys with ICPP under the age of 9 years have been described in the published cohorts to date, accounting for about 8% of all central precocious puberty cases before the age of 9 year and 10% before the age of 10 years (Supplementary Table 1) [21–33]. The incidence of ICPP in boys is around 0.05/10,000 boys under the age of 9 years and 0.24/10,000 boys under the age of 10 years [32, 34, 35], while the incidence of KS is around 15/10,000 births [36]. Thus, the description of 8 cases of KS in around 450 reported cases of ICPP in the general population suggests an “*overrepresentation”* of ICPP in Klinefelter patients. The risk is estimated to be 1/370 KS patients by the Health Register of Belgium [13].

The unexpected “overrepresentation” of ICPP in KS raises two hypotheses. Firstly, while gonadotropin levels in KS patients during ICPP are as high as in 15 years old KS patients without ICPP, the testicular destruction is advanced [17, 37]. Higher gonadotropin levels during ICPP in KS patients compared to those in 46,XY patients suggest that a testicular factor could be a “peripheral initiator of GnRH pulsatility” during precocious puberty that is presumed to be central (ICPP). In KS patients, high estradiol levels is one of the earliest testicular anomalies [38]. Estrogen levels explain the chronological differences in puberty between girls and boys during puberty. Estrogen increases Kiss1 expression (gene coding kisspeptin, a major regulator of GnRH activation) in the hypothalamus during puberty. This effect is exerted through estrogen receptor (Erα). If this process is disrupted, puberty could be delayed [39]. Higher estradiol levels detected early in Klinefelter patients were proposed to precociously activate gonadotropin secretion [28, 31]. Indeed, it has been recently shown that estrogen promotes the expression of genes related to precocious puberty which activate GnRH secretion [40]. Germ cell apoptosis is another early phenomenon in KS [29]. The degree of germ cell apoptosis/seminiferous tissue fibrosis and Leydig cell dysfunction in KS are best reflected by FSH and LH levels respectively. Basal- and stimulated-LH and FSH levels are even higher in the patient with 48,XXYY [12]. This suggests that gonadotropin reserve correlates with the degree of testicular destruction, which is maximal in 48,XXYY patients. This hypothesis is also supported by the description of ICPP cases in patients with Turner syndrome and cryptorchidism [5, 41, 42]. Indeed, the testicular destruction and/or higher estrogen levels in Klinefelter patients would increase the hypothalamic/pituitary gonadotropin reserve and explain why these patients have higher basal and GnRH-stimulated LH and FSH levels during ICPP. These data are interesting and highlight the timing and role of testicular destruction in dysgonosomia to initiate GnRH pulsatility.

Secondly, in our patient, both X chromosomes were of maternal origin. None of the reported cases studied the origin of the supplementary X chromosome. It is worth noting that the supplementary X chromosome is equally inherited from paternal and maternal origin. When the origin of this supplementary X chromosome is maternal, it is hypothesized that some deleterious genes could escape the natural X chromosome inactivation [43, 44]. These deleterious genes could be responsible for an earlier activation of puberty in such patients [17, 45]. In line with this hypothesis, 2 X-linked gene mutations are described in the literature to be associated with ICPP in 2 boys: *NR0B1* (responsible for X-linked adrenal hypoplasia congenita) [46] and *MECP2* (responsible for Rett syndrome) [47]. On the contrary, paternal X chromosome (pX) is associated with further delayed puberty than those with a maternal X chromosome (mX) [17]. Puberty signs with significant increment in LH levels appeared respectively 1.9 and 1.3 years later in patients with pX. Additionally, androgen receptor (AR) gene is located on the X chromosome. It contains CAG repeats in the N terminal domain of exon 1 that could be highly polymorphic. The length of this repetition is inversely correlated with receptor activity [48], and is found to influence clinical presentation of KS [49]. Indeed, shorter CAG repeats and lower AR methylation were linked to premature pubarche [50]. Nevertheless, no study linked CAG repeats or AR activity to precocious puberty in KS patients.

Despite a number of interesting studies investigating early onset of puberty in patients with KS, the genetic basis of idiopathic central precocious puberty in both sexes remains elusive. Further studies using molecular genetics could help understanding the mechanisms of puberty initiation, timing and progression. Specifically, identifying novel X-linked genes in boys with precocious puberty will be instrumental to shed light on the mechanisms underlying puberty initiation.

## Conclusions

ICPP is extremely rare in boys. ICPP patients with KS have higher gonadotropin levels than those with a normal karyotype. This suggests a peripheral or “testicular driver” that amplifies gonadotropin secretion through a positive feedback loop, which in turn triggers the initiation of central PP in such patients. Furthermore, maternal supplementary X chromosome in KS might play a role in the timing of puberty initiation. In boys with ICPP, long term endocrinological follow up until definitive puberty and karyotype analysis are important, specially if gonadotropin levels are significantly elevated or testicular volume is reduced.

## Supplementary Information

**Additional file 1.**

**Additional file 2: Supplementary Table 1.** Incidence of central precocious puberty (ICPP) according to sex. ICPP: Idiopathic central precocious puberty. CPP: Central precocious puberty. ICPP in boys accounts for 10% of the aetiologies of CPP. Data represent number of patients or percentage (%).

## Data Availability

All the data concerning the case are made available in this manuscript.

## Abbreviations

AR: Androgen receptor
CNV: Copy number variations
CPP: Central precocious puberty
FSH: Follicle stimulating hormone
GnRH: Gonadotropin-releasing hormone
hCG: Human chorionic gonadotropin
ICPP: Idiopathic central precocious puberty
KS: Klinefelter syndrome
LH: Luteinizing hormone
LHRH: Luteinizing hormone-releasing hormone
MRI: Magnetic resonance imaging
mX: Maternal X chromosome
PCR: Polymerase chain reaction
PP: Precocious puberty
pX: Paternal X chromosome
SHOX: Short-stature homebox gene
UI: International units

## References

[1] Carel J-C, Léger J (2008). Clinical practice. Precocious puberty N Engl J Med.

[2] Partsch C-J, Heger S, Sippell WG (2002). Management and outcome of central precocious puberty. Clin Endocrinol.

[3] Teilmann G, Pedersen CB, Jensen TK, Skakkebaek NE, Juul A (2005). Prevalence and incidence of precocious pubertal development in Denmark: an epidemiologic study based on national registries. Pediatrics..

[4] Fuqua JS (2013). Treatment and outcomes of precocious puberty: an update. J Clin Endocrinol Metab.

[5] Li L, Gong C (2019). Central precocious puberty as a prelude of gonad dysplasia. Pediatr Investig.

[6] Improda N, Rezzuto M, Alfano S, Parenti G, Vajro P, Pignata C (2012). Precocious puberty in turner syndrome: report of a case and review of the literature. Ital J Pediatr.

[7] Grosso S, Anichini C, Berardi R, Balestri P, Pucci L, Morgese G (2000). Central precocious puberty and abnormal chromosomal patterns. Endocr Pathol.

[8] Wikström AM, Dunkel L (2011). Klinefelter syndrome. Best Pract Res Clin Endocrinol Metab.

[9] Leon N, Wajchenberg BL, Beçak W (1966). Precocious puberty in a case of Klinefelter’s syndrome. Acta Endocrinol.

[10] von Mühlendahl KE, Heinrich U (1994). Sexual precocity in Klinefelter syndrome: report on two new cases with idiopathic central precocious puberty. Eur J Pediatr.

[11] Bertelloni S, Baroncelli GI, Battini R, Saggese G (1996). Central precocious puberty in Klinefelter syndrome: a case report with longitudinal follow-up of growth pattern. Am J Med Genet.

[12] Bertelloni S, Battini R, Baroncelli GI, Guerrini R, Viacava P, Spinelli C (1999). Central precocious puberty in 48,XXYY Klinefelter syndrome variant. J Pediatr Endocrinol Metab JPEM..

[13] Fryns JP, Devriendt K (1997). Precocious puberty in Klinefelter syndrome: non-specific result of neurological deficit?. Am J Med Genet.

[14] Gonzales-Ellis BA, Pingul MM, Reddy S, Boney CM, Wajnrajch MP, Quintos JB (2013). Growth hormone deficiency and central precocious puberty in Klinefelter syndrome: report of a case and review of KIGS database. J Pediatr Endocrinol Metab JPEM..

[15] Pigneur B, Trivin C, Brauner R (2008). Idiopathic central precocious puberty in 28 boys. Med Sci Monit Int Med J Exp Clin Res.

[16] Munns CJF, Haase HR, Crowther LM, Hayes MT, Blaschke R, Rappold G (2004). Expression of SHOX in human fetal and childhood growth plate. J Clin Endocrinol Metab.

[17] Wikström AM, Painter JN, Raivio T, Aittomäki K, Dunkel L (2006). Genetic features of the X chromosome affect pubertal development and testicular degeneration in adolescent boys with Klinefelter syndrome. Clin Endocrinol.

[18] Wikström AM, Dunkel L, Wickman S, Norjavaara E, Ankarberg-Lindgren C, Raivio T (2006). Are adolescent boys with Klinefelter syndrome androgen deficient? A longitudinal study of Finnish 47. XXY boys Pediatr Res.

[19] Aksglæde L, Skakkebæk NE, Almstrup K, Juul A (2011). Clinical and biological parameters in 166 boys, adolescents and adults with nonmosaic Klinefelter syndrome: a Copenhagen experience. Acta Paediatr.

[20] Pacenza N, Pasqualini T, Gottlieb S, Knoblovits P, Costanzo PR, Stewart Usher J (2012). Clinical presentation of Klinefelter’s syndrome: differences according to age. Int J Endocrinol.

[21] Schoevaart CE, Drop SL, Otten BJ, Slijper FM, Degenhart HJ (1990). Growth analysis up to final height and psychosocial adjustment of treated and untreated patients with precocious puberty. Horm Res.

[22] Pescovitz OH, Comite F, Hench K, Barnes K, McNemar A, Foster C (1986). The NIH experience with precocious puberty: diagnostic subgroups and response to short-term luteinizing hormone releasing hormone analogue therapy. J Pediatr.

[23] Klein KO, Barnes KM, Jones JV, Feuillan PP, Cutler GB (2001). Increased final height in precocious puberty after long-term treatment with LHRH agonists: the National Institutes of Health experience. J Clin Endocrinol Metab.

[24] Lebrethon MC, Bourguignon JP (2000). Management of central isosexual precocity: diagnosis, treatment, outcome. Curr Opin Pediatr.

[25] Jaruratanasirikul S, Thaiwong M (2010). Etiologies of precocious puberty: 15-year experience in a tertiary hospital in southern Thailand. J Pediatr Endocrinol Metab JPEM..

[26] De Sanctis V, Corrias A, Rizzo V, Bertelloni S, Urso L, Galluzzi F (2000). Etiology of central precocious puberty in males: the results of the Italian study Group for Physiopathology of puberty. J Pediatr Endocrinol Metab JPEM..

[27] Chemaitilly W, Trivin C, Adan L, Gall V, Sainte-Rose C, Brauner R (2001). Central precocious puberty: clinical and laboratory features. Clin Endocrinol.

[28] Mul D, Bertelloni S, Carel JC, Saggese G, Chaussain JL, Oostdijk W (2002). Effect of gonadotropin-releasing hormone agonist treatment in boys with central precocious puberty: final height results. Horm Res.

[29] Bajpai A, Sharma J, Kabra M, Kumar Gupta A, Menon PSN (2002). Precocious puberty: clinical and endocrine profile and factors indicating neurogenic precocity in Indian children. J Pediatr Endocrinol Metab JPEM.

[30] Shiva S, Fayyazi A, Melikian A, Shiva S (2012). Causes and types of precocious puberty in north-West Iran. Iran J Pediatr.

[31] Soriano-Guillén L, Corripio R, Labarta JI, Cañete R, Castro-Feijóo L, Espino R (2010). Central precocious puberty in children living in Spain: incidence, prevalence, and influence of adoption and immigration. J Clin Endocrinol Metab.

[32] Le Moal J, Rigou A, Le Tertre A, De Crouy-Channel P, Léger J, Carel J-C (2018). Marked geographic patterns in the incidence of idiopathic central precocious puberty: a nationwide study in France. Eur J Endocrinol.

[33] Alikasifoglu A, Vuralli D, Gonc EN, Ozon A, Kandemir N (2015). Changing etiological trends in male precocious puberty: evaluation of 100 cases with central precocious puberty over the last decade. Horm Res Paediatr..

[34] Merke DP, Cutler GB (1996). Evaluation and management of precocious puberty. Arch Dis Child.

[35] Kim YJ, Kwon A, Jung MK, Kim KE, Suh J, Chae HW (2019). Incidence and prevalence of central precocious puberty in Korea: an epidemiologic study based on a National Database. J Pediatr.

[36] Nielsen J, Wohlert M (1991). Chromosome abnormalities found among 34,910 newborn children: results from a 13-year incidence study in Arhus. Denmark Hum Genet.

[37] Wikström AM, Dunkel L (2008). Testicular function in Klinefelter syndrome. Horm Res.

[38] Maqdasy S, Bogenmann L, Batisse-Lignier M, Roche B, Franck F, Desbiez F (2015). Leydig cell tumor in a patient with 49 XXXXY karyotype: a review of literature. Reprod Biol Endocrinol RBE.

[39] Clarkson J, Boon WC, Simpson ER, Herbison AE (2009). Postnatal development of an estradiol-kisspeptin positive feedback mechanism implicated in puberty onset. Endocrinology..

[40] Qian F, Shi N, Zhou H (2020). Estrogen can promote the expression of genes related to precocious puberty in GT1-7 mouse hypothalamic GnRH neuronal cell line via activating G protein-coupled estrogen receptor. Gen Physiol Biophys.

[41] Pajno R, Pacillo L, Recupero S, Cicalese MP, Ferrua F, Barzaghi F (2020). Urogenital abnormalities in adenosine Deaminase deficiency. J Clin Immunol.

[42] Monai E, Johansen A, Clasen-Linde E, Rajpert-De Meyts E, Skakkebæk NE, Main KM (2019). Central precocious puberty in two boys with Prader-Willi syndrome on growth hormone treatment. AACE Clin Case Rep.

[43] Iitsuka Y, Bock A, Nguyen DD, Samango-Sprouse CA, Simpson JL, Bischoff FZ (2001). Evidence of skewed X-chromosome inactivation in 47,XXY and 48,XXYY Klinefelter patients. Am J Med Genet.

[44] Carrel L, Willard HF (2005). X-inactivation profile reveals extensive variability in X-linked gene expression in females. Nature..

[45] Zinn AR, Ramos P, Elder FF, Kowal K, Samango-Sprouse C, Ross JL (2005). Androgen receptor CAGn repeat length influences phenotype of 47,XXY (Klinefelter) syndrome. J Clin Endocrinol Metab.

[46] Shima H, Yatsuga S, Nakamura A, Sano S, Sasaki T, Katsumata N (2016). NR0B1 Frameshift mutation in a boy with idiopathic central precocious puberty. Sex Dev Genet Mol Biol Evol Endocrinol Embryol Pathol Sex Determ Differ.

[47] Tsuji-Hosokawa A, Matsuda N, Kurosawa K, Kashimada K, Morio T (2017). A case of MECP2 duplication syndrome with gonadotropin-dependent precocious puberty. Horm Res Paediatr.

[48] Zitzmann M, Nieschlag E (2003). The CAG repeat polymorphism within the androgen receptor gene and maleness. Int J Androl.

[49] Chang S, Skakkebæk A, Trolle C, Bojesen A, Hertz JM, Cohen A (2015). Anthropometry in Klinefelter syndrome--multifactorial influences due to CAG length, testosterone treatment and possibly intrauterine hypogonadism. J Clin Endocrinol Metab.

[50] Vottero A, Capelletti M, Giuliodori S, Viani I, Ziveri M, Neri TM (2006). Decreased androgen receptor gene methylation in premature pubarche: a novel pathogenetic mechanism?. J Clin Endocrinol Metab.

