# Supplementary material for: Idiopathic central precocious puberty in a Klinefelter patient: highlights on gonadotropin levels and pathophysiology
Source: Basic Clin Androl. 2020 Dec 9;30:19. doi: 10.1186/s12610-020-00117-1 (PMC7724694; doi:10.1186/s12610-020-00117-1)
Supplement: Supplementary file 1 — Additional file 1. [file 12610_2020_117_MOESM1_ESM.docx]

**Supplementary methods**

Chromosome analysis was performed on GTG-banded and RHG-banded metaphases prepared from cultured peripheral blood according to the standard protocols. In order to detect additional submicroscopic copy number aberrations to explain central precocious puberty, microarray-based comparative genomic hybridization (aCGH) was performed on genomic DNA isolated from peripheral blood lymphocytes (Nucleospin® Blood L, Macherey-Nagel, Eurl, Hoerdt, France) using an Agilent 60K oligonucleotide microarray system, according to the manufacturer’s protocol (Agilent 60K Whole Human genome Oligo Microarray, Agilent Technologies, Santa Clara, California, USA). The array was scanned and analyzed with Feature Extraction® 10.5 software (Agilent Technologies). Genomic copy number aberrations were identified using the ADM-II algorithm of DNA analytics® 4.0.76 (Agilent technologies). The results were analyzed using Agilent Cytogenomics 4.03.12 software based on Human Genome Build GRCh37/hg19.

The origin of the supernumerary X chromosome was investigated by fluorescent multiplex PCR studying Short Tandem Repeats polymorphisms (ABI PRISM© Linkage Mapping Set Version 2,5, Applied Biosystems) using microsatellite markers of the X chromosome (DXS987, DXS8091, DXS1214, DXS991, DXS1227, DXS1226, DXS1047, DXS1217, DXS986, DXS1059, DXS8064, DXS8106,DXS8077, DXS1196, DXS1216, DXS8015).

For MKRN3 mutation, the entire coding region and the intron-exon boundaries of *MKRN3* (GenBank accesion number [{"type":"entrez-nucleotide","attrs":{"text":"NC_000015.9","term_id":"224589806","term_text":"NC_000015.9"}}NC_000015.9](https://www-ncbi-nlm-nih-gov.gate2.inist.fr/nuccore/NC_000015.9)) were amplified by PCR followed by automatic sequencing of the products using the Sanger method. Two databases (1000 Genomes and NHLBI EVS) were used to exclude all common variants (minor allele frequency >1%).
