# Supplementary material for: Idiopathic central precocious puberty in a Klinefelter patient: highlights on gonadotropin levels and pathophysiology
Source: Basic Clin Androl. 2020 Dec 9;30:19. doi: 10.1186/s12610-020-00117-1 (PMC7724694; doi:10.1186/s12610-020-00117-1)
Supplement: Supplementary file 2 — Additional file 2: Supplementary Table 1. Incidence of central precocious puberty (ICPP) according to sex. ICPP: Idiopathic central precocious puberty. CPP: Central precocious puberty. ICPP in boys accounts for 10% of the aetiologies of CPP. Data represent number of patients or percentage (%). [file 12610_2020_117_MOESM2_ESM.docx]

**Supplementary Table 1:** Incidence of central precocious puberty according (CPP) to sex.

|  |  | Girls | | Boys | |
| --- | --- | --- | --- | --- | --- |
| References | **Period** | **Idiopathic** | **Neurogenic** | **Idiopathic** | **Neurogenic** |
| *Schoevaart et al.* [21] | *1965-1979* | 23 | 4 | 7 | 0 |
| *Pescovitz et al.* [22] | *1979-1983* | 60 | 27 | 2 | 18 |
| *Klein et al.* [23] | *1979-2001* | 54 | 26 | 3 | 15 |
| *Lebrethon et al.* [24] | *1989-1997* | 108 | 27 | 3 | 4 |
| *Jaruratanasirikul et al.* [25] | *1995-2009* | 58 | 10 | 0 | 5 |
| *Da Sanctis et al.* [26] | *2000* | - | - | 27 | 18 |
| *Chemaitilly et al.* [27] | *2001* | 186 | 44 | 7 | 19 |
| *Mul et al.* [28] | *2001* | - | - | 14 | 12 |
| *Bajpai et al.* [29] | *2002* | 61 | 16 | 8 | 10 |
| *Shiva et al.* [30] | *2007-2009* | 34 | 4 | 1 | 1 |
| *Soriano-Guillén et al.* [31] | *2008-2010* | 201 | 25 | 16 | 8 |
| *Le Moal et al.* [32] | *2011-2013* | 3519 | - | 352 | - |
| *Alikasifoglu et al.* [33] | *2003-2014* | *-* | *-* | 74 | 26 |
| Total according to sex | | **4304/4487**  **(96%)** | **183/4487**  **(4%)** | **514/650**  **(79%)** | **136/650**  **(21%)** |
| Total for all cases of CPP | | **83.7%** | **3.6%** | **10%** | **2.7%** |

ICPP: Idiopathic central precocious puberty. CPP: Central precocious puberty. ICPP in boys accounts for 10% of the aetiologies of CPP. Data represent number of patients or percentage (%).
